# Micronutrient Deficiencies in Heart Transplant Recipients—Scoping Review

**DOI:** 10.3390/nu18101485

**Published:** 2026-05-07

**Authors:** Maja Ławniczek, Julia Habryka, Sabina Krupa-Nurcek

**Affiliations:** 1 Faculty of Medicine, Collegium Medicum, University of Rzeszów, 35-310 Rzeszow, Poland; maajanka@gmail.com (M.Ł.); julhab99@gmail.com (J.H.); 2 Department of Surgery, Faculty of Medicine, Collegium Medicum, University of Rzeszów, 35-310 Rzeszow, Poland

**Keywords:** micronutrient, deficiencies, heart transplant, recipients

## Abstract

**Background/Objectives:** Heart transplant recipients are particularly at risk for micronutrient deficiencies due to chronic immunosuppression, metabolic disorders, gastrointestinal absorption disorders, and increased postoperative demand. Despite a growing body of evidence suggesting their clinical relevance, the prevalence, characteristics, and consequences of these deficiencies remain poorly defined. The aim of this review was to assess of selected micronutrient deficiencies in personnel after heart vaccination and risk factors for their control. **Methods:** This scoping review was conducted in accordance with the Joanna Briggs Institute’s scope review methodology and presented in accordance with the PRISMA-ScR guidelines. A systematic search of PubMed, Scopus, EBSCO, Web of Science, Google Scholar, and Cochrane Library (January–February 2026) identified studies assessing micronutrient deficiencies in adult heart transplant recipients. Original publications, meta-analyses, and reviews available in full text in English were eligible for the review. Data extraction was carried out independently by two reviewers; using the PCC (Population–Concept–Context) model. **Results:** Of the 35 pre-identified records, 12 studies met the inclusion criteria. The most commonly reported deficiencies included iron, vitamin D, and B vitamins, and their incidence varied widely due to heterogeneous diagnostic criteria. Iron deficiency—both absolute and functional—was common and often associated with inflammation and impaired hepcidin regulation. Vitamin D deficiency persisted before and after transplantation and was associated with impaired bone health, inflammation, and a potentially increased risk of infection. Elevated homocysteine levels associated with low levels of folic acid and vitamin B6 have been identified as potential contributing factors to atherosclerotic and thrombotic complications. Limited evidence also points to deficiencies in iodine, zinc, and other trace elements. **Conclusions:** Micronutrient deficiencies are common among heart transplant recipients and can adversely affect immune system function, cardiovascular risk, and overall clinical outcomes. Routine evaluation and targeted correction of deficiencies should be considered in post-transplant care. Further prospective, multicenter, and interventional studies are needed to establish standardized diagnostic criteria and evidence-based supplementation strategies.

## 1. Introduction

Heart transplantation remains the therapy of choice for end-stage heart failure, significantly improving patient survival and quality of life [1,2]. Advances in immunosuppression, perioperative care, and transplant rejection monitoring have contributed to a significant reduction in early mortality, but long-term metabolic and nutritional complications remain a significant clinical challenge [3]. Among them, special attention is paid to micronutrient deficiencies, which can affect the functioning of the immune system, the metabolism of immunosuppressive drugs, the risk of infection, and the development of cardiovascular complications. Heart transplant patients are at increased risk of eating disorders due to a number of causes, including chronic immunosuppression, changes in intestinal absorption, drug interactions, dietary restrictions, and increased metabolic demand during the recovery period [4]. Previous studies indicate frequent deficiencies of vitamin D, magnesium, iron, zinc or B vitamins, but the data remain heterogeneous and the scale of the problem may be underestimated. There is also a lack of consistent guidelines for routine evaluation and supplementation of micronutrients in this population. Understanding the micronutrient deficiency profile of heart transplant recipients is critical to optimizing postoperative care, improving prognosis, and reducing complications associated with both immunosuppression and comorbidities [2,3].

In the general population, these deficiencies can lead to hematological, neurological, bone, or immune disorders, while in heart transplant recipients their importance is due to chronic immunosuppression, increased risk of infection, wound healing disorders, drug interactions affecting absorption, and altered metabolic demand after surgery [1,3,4]. As a consequence, even subclinical deficiencies can have significant clinical implications. Early identification and correction of deficiencies can reduce the risk of infection, improve immunosuppression tolerance, support tissue regeneration, reduce the risk of cardiovascular complications, and improve overall nutrition and quality of life [5,6]. Despite this, routine micronutrient monitoring is not standard in all transplant centers, and guidelines remain inconsistent [5,7].

Micronutrients—vitamins, trace elements and electrolytes—play a key role in regulating the immune response, antioxidant processes, energy metabolism, and tissue regeneration, which is particularly important in heart transplant recipients exposed to chronic immunosuppression, inflammation, and increased metabolic demand. Their deficiencies can impair both innate and acquired immunity, as many of them act as essential cofactors for lymphocyte proliferation, cytokine synthesis, and normal neutrophil and NK cell activity. Vitamin D modulates the expression of antibacterial peptides and promotes the formation of an anti-inflammatory profile, while zinc is essential for the maturation of T lymphocytes in the thymus and the stabilization of cytokine signaling pathways. Iron, although crucial for the functioning of immune cells, is dysregulated in conditions of chronic inflammation, which can further weaken the immune response [3].

Many micronutrients also participate in antioxidant protection. Selenium is a component of glutathione peroxidase, zinc acts as a superoxide dismutase cofactor, and vitamin D affects the regulation of pro-inflammatory cytokines, reducing oxidative stress exacerbated by immunosuppressive therapy. Deficiencies in these components can lead to increased production of reactive oxygen species and cellular damage [3,4].

Micronutrients are also essential for proper energy metabolism. Iron and B vitamins are involved in mitochondrial energy production, the Krebs cycle, and β-oxidation of fatty acids, and their deficiencies can manifest as fatigue, reduced exercise tolerance, and slowed recovery after transplantation. Additionally, zinc, iron, and vitamin D play a vital role in collagen synthesis, angiogenesis, and cell proliferation, making them crucial for postoperative wound healing and long-term tissue regeneration. Due to these multidirectional functions, even subclinical micronutrient deficiencies can negatively affect immunity, metabolic stability, and repair processes in heart transplant recipients, highlighting the need for systematic monitoring and targeted supplementation in transplant care [5,7].

Significant geographical differences are also observed in the population of cardiac recipients, resulting from different nutritional practices, different availability of micronutrient preparations, and different strategies for monitoring hematologic and biochemical parameters [3]. In Europe, where access to transplant care and supplementation is relatively wide, unified protocols for assessing nutritional status are more often used, but there are still differences between Western European countries and Central and Eastern European countries, especially in the diagnosis of iron, vitamin D, or B vitamin deficiency which may affect the higher incidence of deficiencies even before transplantation. These differences are further exacerbated by different models of postoperative care, the availability of laboratory testing, and local guidelines for supplementation [3].

**Figure 1:**
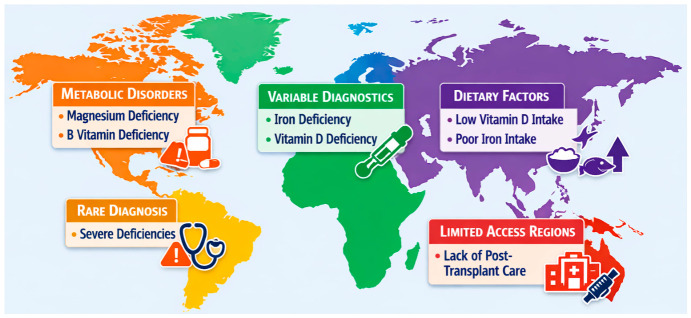
Global differences in micronutrient deficiencies in cardiac recipients, highlighting key factors for individual continents.

An important factor differentiating the populations of cardiac recipients on different continents is also the immunosuppression regimens used, which affect metabolism, absorption, and the need for micronutrients. In North America, intensive immunosuppressive protocols are more commonly used, which may increase the risk of metabolic disorders and deficiencies, especially of magnesium, zinc, or B vitamins. In regions with limited access to specialist transplant care, such as parts of South America or South Asia, micronutrient deficiencies may be more severe and less frequently diagnosed, further increasing the risk of complications after transplantation [4]. These intercontinental differences highlight the need for analyses that take into account the local clinical context and the need to develop more harmonized standards for the monitoring and treatment of micronutrient deficiencies in cardiac recipients. Figure 1 shows global differences in micronutrient deficiencies in cardiac recipients, highlighting key factors for individual continents.

The aim of this study was to assess of selected micronutrient deficiencies in personnel after heart vaccination and risk factors for their control.

## 2. Materials and Methods

### 2.1. Study Design

We conducted a scoping review because our aim was to assess of selected micronutrient deficiencies in personnel after heart vaccination and risk factors for their control. Currently, there is little data on the choice between systematic review and the scope of the review during data synthesis. This is common in a situation where the literature has not previously been comprehensive or when it is extensive or complex and heterogeneous, making it difficult to classically control [8].

Our review was prepared with a methodology developed by the Joanna Briggs Institute and based on a database in preferred reporting items for systematic reviews and meta-analyses for scope reviews (PRISMA-ScR) [9,10].

### 2.2. Inclusion and Exclusion Criteria

We developed a research question that clearly defined the population, conceptual framework, and context of the review. This helped identify important aspects related to the phenomenon of deficiencies of selected micronutrients of the control in heart transplant recipients and to identify the risk factors affecting their functioning.

Inclusion criteria included: all published papers; original articles (both observational and randomized), meta-analyses, systematic and narrative reviews; publications available in full text; articles written in English.

Exclusion criteria included: case reports, comments, letters to the editor, book chapters; works without access to the full text; articles published in a language other than English.

#### 2.2.1. Population

The review included studies describing the consequences of deficiencies in selected micronutrients of the control group in heart transplant recipients and identifying the risk factors affecting their functioning. In this review, micronutrients were defined as a specific vitamins and trace elements—such as iron, vitamin D, B vitamins, zinc, selenium, and iodine—that play essential roles in immune regulation, metabolic stability and graft function, and whose deficiencies are particularly common and clinically relevant in heart transplant recipients [1,2,3,5].

#### 2.2.2. Concept

The focus was on the consequences of deficiencies in selected micronutrients of the control group in patients after heart transplantation and on identifying the risk factors affecting their functioning. The aim of this study was to assess of selected micronutrient deficiencies in personnel after heart vaccination and risk factors for their control.

#### 2.2.3. Context

The studies included in this review were conducted in the clinical context of adult patients after heart transplantation. The analysis covered research performed in various transplant centers and healthcare systems, reflecting differences in immunosuppressive regimens, nutritional monitoring practices, and post-transplant care models. The context also encompassed both early and long-term post-transplant periods, during which patients are exposed to chronic immunosuppression, metabolic alterations, gastrointestinal absorption disturbances, and increased vulnerability to micronutrient deficiencies. This setting allowed for the identification of the consequences of selected micronutrient deficiencies and the risk factors contributing to their development in heart transplant recipients.

#### 2.2.4. Types of Studies

This review covered a wide range of research projects, not just retrospective observational studies.

### 2.3. Search Strategy

A comprehensive literature search was conducted in six electronic databases: PubMed, Scopus, Web of Science, EBSCO, Cochrane Library, and Google Scholar. The search covered the period from 10 January 2026 to 20 February 2026, as specified in the protocol. The strategy was developed in accordance with the PRISMA-ScR and JBI guidelines to ensure transparency and reproducibility.

The search combined controlled vocabulary (e.g., MeSH terms in PubMed) and free-text keywords related to micronutrients, deficiencies, and heart transplantation. Filters were applied to restrict results to adult human populations, English-language publications, and full-text availability. Eligible study types included observational studies, randomized controlled trials, systematic reviews, narrative reviews, and meta-analyses. Below is the full search strategy used in PubMed, provided as an example of the reproducible search strings applied across databases.

For Scopus, Web of Science, EBSCO, Cochrane Library, and Google Scholar, the search strategy was adapted to the indexing structure of each database. Boolean operators (AND/OR), truncation symbols (*), and controlled vocabulary (where applicable) were used to ensure comprehensive retrieval of relevant studies. The core search concepts remained consistent across platforms:

Concept 1: micronutrients (iron, vitamin D, B vitamins, zinc, iodine, trace elements)
Concept 2: heart transplantation
Concept 3: deficiency, insufficiency, metabolic disturbance

The complete list of retrieved records, screening decisions, and reasons for exclusion is presented in the PRISMA-ScR flow diagram. Table 1 shows the summary of search strategy.

### 2.4. Extraction of Data

A form prepared in accordance with the JBI guidelines on scoping reviews [9], including key information from the analyzed publications, was used to compile the data. The data extraction process—referred to in scoping reviews as “data plotting” [10,11]—was carried out independently by two reviewers. The Population–Concept–Context (PCC) scheme was used to identify relevant studies. The data extraction process was carried out in accordance with the Joanna Briggs Institute (JBI) guidelines for scoping reviews to ensure consistency and repeatability of the procedure. A standardized data extraction form was used, developed on the basis of JBI recommendations and adapted to the specificities of this review. The form included: bibliographic data, population characteristics, micronutrients evaluated, deficiency definitions used, diagnostic methods, outcomes, and risk factors. Before the full extraction began, pilot testing of the form was carried out on randomly selected publications to ensure that all relevant elements were recorded in an unambiguous and consistent manner. At this stage, the necessary adjustments were made to the form. The data extraction was carried out by two independent reviewers who had previously calibrated by extracting data from three pilot articles in parallel. The purpose of the calibration was to ensure consistency in the interpretation of the form fields and to minimize the risk of discrepancies in the further stage of work. After obtaining satisfactory compliance, the reviewers proceeded to a full data extraction. Any differences in the extracted information were resolved through discussion and consensus; if necessary, a third reviewer was consulted. According to the scoping review methodology, no formal compliance indicators (e.g., kappa coefficient) were calculated because the purpose of the review was to map the available evidence rather than to assess the methodological quality of the studies.

### 2.5. Critical Appraisal Process

A scoping review may include a review of current evidence without including a methodological assessment of the included studies [9].

### 2.6. Process for Including Publications to the Review

**Figure 2:**
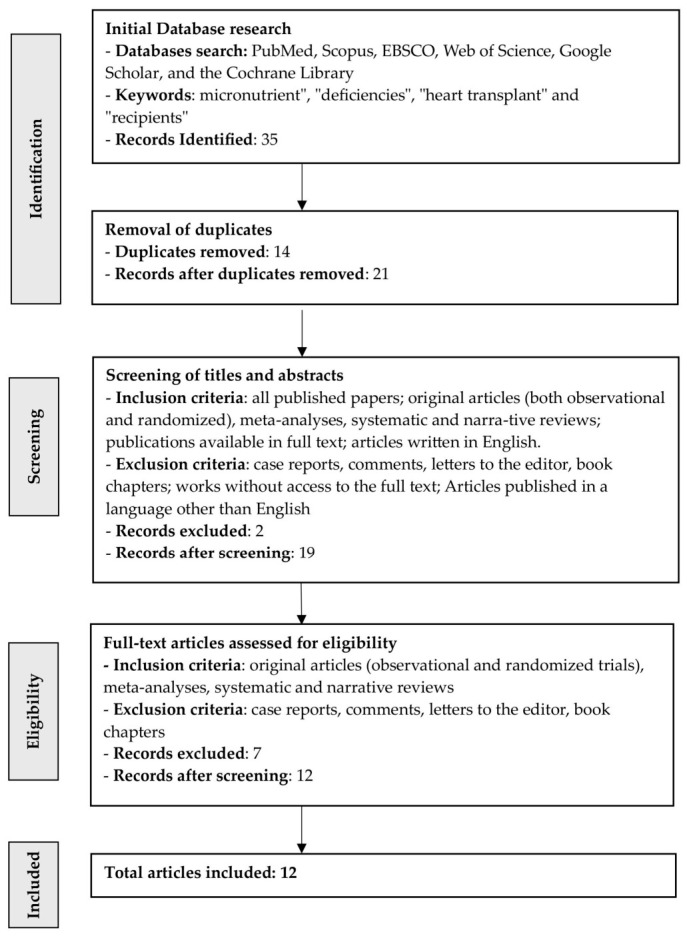
Literature search and selection flowchart for this review.

Our scoping review initially identified 35 articles, 12 of which were ultimately included in the analysis of the consequences of deficiencies in selected micronutrients of the control group in patients after heart transplantation and to identify the risk factors affecting their functioning (Figure 2). After removing duplicates (n = 14), 21 articles remained. After reviewing the articles according to the inclusion and exclusion criteria (n = 2), 19 articles remained. Seven publications did not provide full text and were excluded. As a result, after meeting all requirements, 12 publications were included in the review. The studies were conducted in Poland (n = 6), USA (n = 3), Norway (n = 2), and Austria (n = 1). The results are presented in Table 2.

### 2.7. Selection Process

The selection process of the publication was carried out in accordance with the PRISMA-ScR guidelines to ensure transparency and minimize the risk of selection error. All records retrieved from the databases were imported into the bibliography management program, and duplicates were removed automatically and manually.

Two independent reviewers selected articles at each stage. First, an independent review of titles and abstracts was carried out, followed by the full texts of publications qualified for further evaluation.

The selection was made completely independently, and any discrepancies between the reviewers were resolved through discussion and consensus. In situations requiring additional opinion, a third reviewer was consulted.

According to the scoping review methodology recommended by JBI, no formal inter-reviewer agreement rates (e.g., kappa ratio) were calculated because the purpose of the review was to map the available evidence rather than to assess the methodological quality of the research. The selection process is illustrated in a flow diagram compliant with PRISMA-ScR.

The selection process was carried out in accordance with the PRISMA-ScR guidelines and included four stages:

1. Identification

All records obtained from databases (PubMed, Scopus, Web of Science, EBSCO, Cochrane Library, Google Scholar) were imported into the bibliography management program. Duplicates were removed automatically and manually.

2. Screening (Pre-Selection)

Two independent reviewers conducted an initial review of the titles and abstracts, using predefined inclusion and exclusion criteria. Publications that did not meet the criteria were excluded at this stage.

3. Eligibility (Evaluation of full texts)

The full texts of the articles qualified after the screening stage were evaluated independently by two reviewers. Any discrepancies regarding the qualifications of publications were resolved through discussion and consensus; if necessary, a third reviewer was consulted.

4. Inclusion

Publications that met all PCC criteria were eventually included in the review. The number of publications at each stage is shown in a flow diagram compliant with PRISMA-ScR.

### 2.8. Selection of Sources of Evidence Section

Two independent reviewers carried out the selection at each stage, the selection was carried out independently, discrepancies were resolved through discussion and consensus, a third reviewer was consulted if necessary, and the process is described in accordance with PRISMA-ScR and JBI guidelines.

## 3. Major Nutrient and Micronutrient Deficiencies in Heart Transplant Recipients

**Figure 3:**
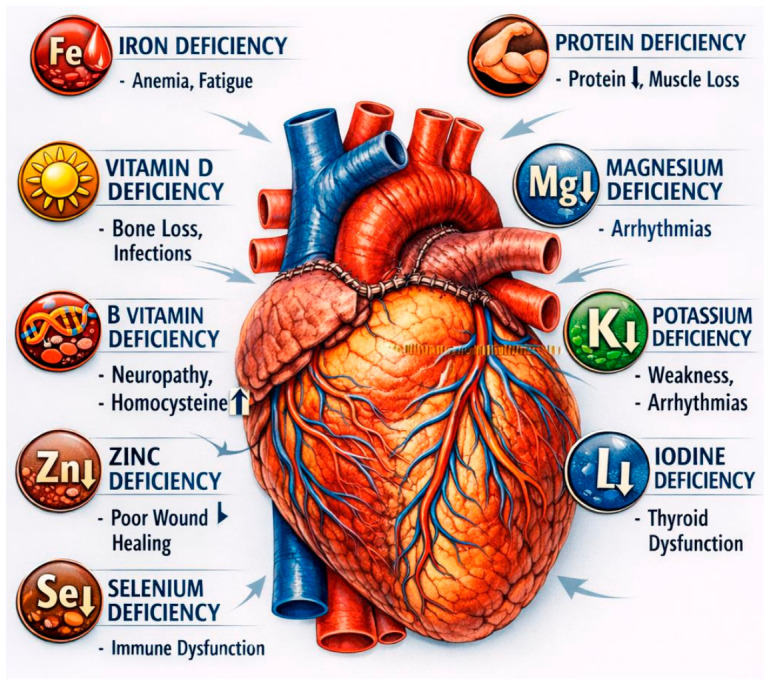
Micro- and macronutrient deficiencies in heart recipients (a down arrow indicates a decrease in value, and an up arrow indicates an increase in value).

Macro- and micronutrient deficiencies are a significant and often underestimated problem in the long-term care of heart transplant recipients [4,24,25,26]. Complex metabolic changes, chronic inflammation, multidrug use, and long-term use of immunosuppressive therapy create a specific physiological environment that predisposes this group of patients to the occurrence of both overt and subclinical eating disorders [27]. International studies clearly indicate that deficiencies of key vitamins, trace elements, and energy components are common after transplantation and may contribute to weakened immunity, shortened transplant survival, increased susceptibility to infections, and accelerated development of cardiovascular and metabolic complications [24,27,28,29]. Along with the improvement of survival after heart transplantation, the importance of a comprehensive assessment of nutritional status and targeted supplementation, which are becoming an integral element of modern care for transplant recipients, is growing [30,31]. Figure 3 shows micro- and macronutrient deficiencies in heart recipients.

### 3.1. Iron Deficiency in Heart Recipients

Iron deficiency is one of the most commonly underestimated metabolic disorders in heart recipients, even though its consequences can significantly affect quality of life, exercise tolerance, immune system function, and overall prognosis after transplantation [32]. In recent years, there has been a growing interest in this issue, which results both from the progress in understanding the pathophysiology of micronutrient deficiency in organ transplant patients and from the increasing number of international reports indicating a high incidence of iron deficiency in this population [12,13,14]. Unlike the general population or patients with heart failure prior to transplantation, heart recipients are a special group—burdened with chronic immunosuppression, increased risk of infection, malabsorption and chronic inflammation, which may modulate iron metabolism in a different way than in other clinical conditions [19]. Data from international studies indicate that iron deficiency occurs in 30–60% of heart recipients, depending on the diagnostic criteria used, the time since transplantation, and the characteristics of the study population. In European studies, this frequency oscillates around 40%, while in North American analyses it is even higher, especially among patients with chronic transplant dysfunction or concomitant kidney disease [21,33,34,35]. It is worth noting that iron deficiency can occur both with and without anemia, which makes it even more difficult to diagnose it in everyday clinical practice. Iron deficiency can indeed occur both in overt (with anemia) and latent (without anemia), which has important diagnostic consequences. In clinical practice, more than half of the cases of iron deficiency proceed without a decrease in hemoglobin concentration, because the body compensates for the deficit for a long time by mobilizing iron stores from ferritin and increasing the efficiency of its use in erythropoiesis. In inflammation, which is common in transplant recipients, the situation becomes even more complex. Ferritin acts as an acute phase protein, so its concentration can be normal or elevated despite the actual iron deficiency [34]. At the same time, hepcidin, the level of which increases in response to inflammation, inhibits the release of iron from macrophages and enterocytes, leading to the so-called functional iron deficiency. In this situation, iron is present in the body, but unavailable for erythropoiesis, which may not cause an immediate decrease in hemoglobin, but leads to clinical symptoms and deterioration of exercise tolerance. For this reason, the diagnosis of iron deficiency in transplant patients requires an evaluation of a full panel of iron metabolism parameters, including TSAT, ferritin, CRP, and, if possible, hepcidin, and not just hemoglobin concentration [34]. Significant geographical differences are also observed among cardiac transplant recipients, resulting from factors such as diverse dietary practices, variable availability of iron preparations, differences in strategies for monitoring hematologic parameters, and heterogeneity in immunosuppressive regimens. Other studies suggest a slightly lower incidence of iron deficiency, which may be due to a different dietary profile, while in European and American populations this problem appears to be more severe, especially in patients with long-term immunosuppression with calcineurin inhibitors [19,21,32]. The mechanisms leading to iron deficiency in heart recipients are complex and multifactorial. Although oral supplementation is standard in the general population, its effectiveness in cardiac recipients is limited. This is due to malabsorption disorders, drug interactions, and frequent side effects from the gastrointestinal tract. European studies indicate that the improvement in iron parameters after oral supplementation is small and often short-lived. In recent years, there has been a growing interest in intravenous iron preparations, such as iron carboxymaltose or isomaltose iron [34,35,36,37]. International studies have shown that they improve exercise tolerance, increase VO_2_peak, reduce symptoms of fatigue, improve quality of life, are well tolerated, and are safe in heart recipients. Although data are still limited, the results of pilot and observational studies are promising and suggest that intravenous supplementation may become the standard of practice in this population [23].

#### Summary Related to Iron Deficiency in Heart Recipients

Iron deficiency is a common and at the same time underestimated problem in heart recipients. Its pathophysiology is complex and includes both classical mechanisms of deficiency and disorders resulting from chronic inflammation and immunosuppression [21]. Clinical consequences are significant and include reduced exercise tolerance, poorer quality of life, increased risk of hospitalization, and potentially worse prognosis. Diagnosis requires the use of criteria that take into account the impact of inflammation, and treatment—especially intravenous supplementation—seems to be a promising therapeutic strategy [35,36,37,38]. In light of the available data, iron deficiency should be routinely monitored and actively treated in heart recipients, which may contribute to improving their functioning and quality of life [38].

### 3.2. Vitamin D

Vitamin D, traditionally associated with the regulation of calcium-phosphate metabolism and bone health, has become the subject of intensive research in the context of immunology, chronic diseases, and transplantology in the last two decades [39]. In heart transplant recipients, its role is of particular importance, as this population is characterized by an extremely high risk of metabolic disorders, infections, immune complications, and adverse changes in the cardiovascular system. International studies indicate that both vitamin D deficiency and excess can affect post-transplant treatment outcomes, although the mechanisms of these relationships are complex and are still being studied intensively [15,16,40].

Vitamin D deficiency is common in the general population; however, it is much more common and clinically important in heart transplant recipients. European and North American studies indicate that up to 70–90% of patients waiting for a heart transplant have a concentration of 25(OH)D below the values considered optimal. After transplantation, the situation often does not improve, and in some patients there is even a further decrease in vitamin D levels [41].

The main causes of deficiency include limited exposure to sunlight, resulting from a severe clinical condition and long-term hospitalizations; chronic heart failure, which is associated with impaired absorption and metabolism of vitamin D; the use of immunosuppressive drugs, especially glucocorticoids, which accelerate the catabolism of vitamin D; renal failure, often co-occurring in patients with advanced heart failure; and obesity, which favors sequestration vitamin D in adipose tissue [20]. Other studies confirm that vitamin D deficiency is one of the most common metabolic disorders in this group of patients and can persist for many months after transplantation if not properly treated.

Vitamin D has an immunomodulatory function, affecting both innate and acquired immunity. Vitamin D receptors (VDRs) are found on T cells, B cells, macrophages, and dendritic cells [15,20]. The active form of vitamin D—1,25(OH)_2_D—inhibits the differentiation of Th1 and Th17 lymphocytes, while supporting the development of regulatory T cells. This mechanism is particularly important in heart transplant recipients whose immune balance determines the risk of transplant rejection. International observational studies suggest that low vitamin D levels may be associated with a higher incidence of acute rejection episodes, an intensification of the inflammatory response, a higher risk of bacterial and viral infections, including CMV (Cytomegalovirus) and EBV (Epstein–Barr Virus), and a worse overall prognosis [42]. Some studies do not support the direct effect of vitamin D deficiency on transplant rejection, suggesting that vitamin D may be a marker of overall health rather than a causal factor. Nevertheless, the role of vitamin D as a potential modulator of the immune response remains the subject of intensive clinical trials. Other studies have shown that low vitamin D levels correlate with a higher risk of developing transplant vascular disease (TVD), one of the most serious complications late after heart transplantation. TVD is an immuno-inflammatory process, and vitamin D can modulate its course by affecting pro-inflammatory cytokines and immune system cells [16,20,41].

Other European studies have shown that vitamin D supplementation in heart transplant recipients may reduce bone loss in the first year after transplantation, although these effects are moderate and depend on the dose and baseline concentration of 25(OH)D. Vitamin D supplementation in combination with bisphosphonates has been shown to be more effective than vitamin D alone in several randomized trials. which suggests that her role is supportive rather than dominant. Excess vitamin D is most often the result of uncontrolled supplementation, less often due to metabolic disorders [42,43]. Studies indicate that in heart transplant patients, even moderate exceeding of recommended doses can lead to toxicity, as vitamin D metabolism is impaired by immunosuppressants and concomitant kidney disease. Vitamin D may affect the metabolism of certain immunosuppressive drugs, especially calcineurin inhibitors (tacrolimus, ciclosporin) [20,39]. In vitro studies suggest that vitamin D may modulate the activity of cytochrome P450 enzymes, potentially affecting drug concentrations. However, clinical data are limited and inconclusive.

On the other hand, glucocorticoids accelerate the degradation of vitamin D, which can exacerbate vitamin D deficiency [22]. Antifungal medications, such as ketoconazole, can inhibit vitamin D metabolism, increasing the risk of toxicity. For this reason, monitoring of 25(OH)D levels in heart transplant recipients should be carried out regularly, especially in the first year after transplantation. Infections are one of the leading causes of morbidity and mortality after heart transplantation [41,42,43]. Vitamin D plays a role in the activation of antibacterial peptides, such as cathelicidin, and in the regulation of the inflammatory response [44]. Studies have shown that low levels of vitamin D may be associated with a higher risk of respiratory infections, TVD infections, and opportunistic infections [39].

The results of interventional studies are inconclusive. Some studies suggest that vitamin D supplementation may reduce the risk of infection, while others show no significant differences. It is possible that vitamin D only acts as a marker of overall health and not as a causal factor [45,46].

### 3.3. Homocysteine

Homocysteine, which is a sulfur amino acid produced as a result of methionine metabolism, has been the subject of interest of researchers dealing with cardiovascular diseases, nephrology, and transplantology for many years. Its concentration in plasma is the result of genetic, nutritional, metabolic, and pharmacological factors [47]. In heart transplant recipients, the problem of hyperhomocysteinemia is particularly important, as this population is characterized by an increased risk of cardiovascular complications, metabolic disorders, kidney dysfunction, and chronic inflammation. International studies indicate that both homocysteine deficiency and excess can affect post-transplant treatment outcomes, although the mechanisms of these relationships are complex and are still being studied intensively [48]. In recent years, increased attention has been paid to the role of homocysteine as a risk biomarker, a potential pathogenetic factor, and an element that can be modified through nutritional and pharmacological interventions. Hyperhomocysteinemia is much more common in heart transplant recipients than in the general population. Studies conducted in various countries have shown that elevated homocysteine levels are observed in 40–70% of heart transplant patients, and in some of them the values exceed the threshold considered to be a significant risk factor for cardiovascular complications [20]. The reason for such a high incidence of hyperhomocysteinemia is a combination of factors: chronic kidney failure, the use of immunosuppressive drugs, deficiencies of B vitamins, and intestinal absorption disorders, as well as chronic inflammation and oxidative stress [19]. Many studies emphasize that kidney function is one of the most important determinants of homocysteine concentrations, and in heart transplant recipients, kidney dysfunction is a common complication associated with calcineurin inhibitor therapy. For this reason, hyperhomocysteinemia can be both a marker and a potential factor in worsening kidney function, creating a metabolic vicious cycle [49]. Homocysteine is associated with numerous pathophysiological mechanisms that can affect the health of heart transplant recipients. International studies have shown that elevated homocysteine levels promote endothelial dysfunction, increase oxidative stress, intensify inflammatory processes, and promote the proliferation of vascular smooth muscle cells [48,50]. These mechanisms are particularly important in the context of cardiac allograft vasculopathy (CAV), which is one of the most serious late complications after heart transplantation. CAV is an immunoinflammatory process in which the inner membrane of the coronary vessels of the transplanted heart is thickened. Other studies have shown that patients with higher homocysteine levels have a higher risk of CAV progression, although not all studies support this relationship [51]. Some researchers suggest that homocysteine may act as a marker of overall metabolic and inflammatory load, rather than as a direct causative factor. Nevertheless, its role in the pathogenesis of CAV remains the subject of intensive research. Hyperhomocysteinemia is also associated with a higher risk of thrombotic complications. In heart transplant recipients, this risk is increased due to the use of immunosuppression, lipid disorders, hypertension, and renal dysfunction. Homocysteine can affect the coagulation system by increasing the activity of prothrombotic factors, inhibiting protein C and S, and damaging the endothelium [49,51,52,53]. Studies have also shown that heart transplant patients with hyperhomocysteinemia are more prone to deep vein thrombosis and embolic events, although these data are inconclusive and require further analysis. Some studies have shown no significant association between homocysteine levels and thrombosis risk, suggesting that its role may be modulated by other factors such as kidney function, medications used, and general inflammation [52].

Homocysteine deficiency, although much rarer than its excess, can also be a clinical problem. Too low homocysteine levels may be associated with malnutrition, severe malabsorption, liver disease, or excessive supplementation of B vitamins. Homocysteine deficiency may also indicate disorders in methionine metabolism, which may affect protein synthesis, immune system function, and regenerative processes. Several studies have shown that very low homocysteine levels may be associated with a worse prognosis in patients with severe chronic diseases, although data on heart transplant recipients are limited. Homocysteine interactions with immunosuppressive drugs are another important area of research. Calcineurin inhibitors, such as tacrolimus and ciclosporine, may affect homocysteine metabolism by influencing renal function and enzymes involved in its metabolism [48,51,52,53,54,55]. Glucocorticoids, in turn, can affect the metabolism of B proteins and vitamins, which indirectly affects homocysteine concentrations. Antiproliferative drugs such as mycophenolate mofetil can interfere with intestinal absorption, which can also affect homocysteine levels. Several studies have shown that patients treated with tacrolimus have higher homocysteine levels than patients treated with ciclosporin, although these data are inconclusive and may depend on kidney function and the dose of medication [56].

Homocysteine can also affect the immune system, which is especially important in heart transplant recipients. In vitro studies have shown that elevated homocysteine levels may potentiate the production of pro-inflammatory cytokines such as IL6 and TNFα and affect the activation of T cells. Several observational studies have shown that patients with higher homocysteine levels have a higher frequency of rejection episodes, but other studies do not support this relationship [51,53]. It is possible that homocysteine acts as a marker of general inflammation rather than a direct pathogenetic factor. In the context of infections, which are one of the main causes of morbidity and mortality after heart transplantation, the role of homocysteine is less clear. Some studies suggest that hyperhomocysteinemia may be associated with a higher risk of infection, especially in patients with renal dysfunction; however, this data is limited [55]. It is possible that homocysteine affects endothelial and microvascular function, which may interfere with the immune response, but these mechanisms require further research. An important clinical aspect is the possibility of modifying homocysteine concentrations through nutritional and pharmacological interventions. Folic acid, vitamin B6, and vitamin B12 supplementation is effective in lowering homocysteine levels, but its effect on the clinical prognosis in heart transplant recipients remains unclear. In many centers, routine supplementation of B vitamins is used in patients with hyperhomocysteinemia, especially in those with renal dysfunction [56]. However, the need to individualize therapy is increasingly emphasized, because excessive supplementation can lead to too low homocysteine concentration, which can also be unfavorable. The international literature emphasizes the need for further research on the role of homocysteine in heart transplant recipients. There is a lack of large randomized trials assessing the impact of homocysteine reduction on the risk of CAV, transplant rejection, or thrombotic complications [57,58]. Research is also needed on the interactions of homocysteine with immunosuppressive drugs and on the role of homocysteine as a biomarker of renal function and overall metabolic status. It is still not known whether homocysteine is a causal factor or just a risk marker, but its importance in transplantology remains indisputable [59].

### 3.4. B Vitamins

B vitamins play a key role in numerous metabolic processes, including DNA synthesis, nervous system function, energy metabolism, regulation of the immune response, and maintenance of normal homocysteine levels [60]. Heart transplant recipients are particularly important because this population is at increased risk of metabolic disorders, malnutrition, kidney dysfunction, chronic inflammation, and cardiovascular complications [17,60]. International studies indicate that both deficiency and excess of B vitamins can affect the outcome of treatment after heart transplantation, although the mechanisms of these relationships are complex and still intensively studied. In recent years, increased attention has been paid to the role of vitamins B6, B9 (folic acid), and B12, as their deficiency is most commonly observed in transplant patients and is of the greatest clinical importance. However, other vitamins in this group—such as thiamine (B1), riboflavin (B2), niacin (B3), or biotin (B7)—may also play an important role in the metabolism and functioning of the transplant recipient’s body [17].

In heart transplant recipients, B vitamin deficiencies are common and result from many factors. Studies conducted in various countries have shown that up to 30–60% of heart transplant patients are deficient in one or more B vitamins, especially folic acid and vitamin B12. The cause is both increased metabolic demand and intestinal absorption disorders, which may result from the chronic use of immunosuppressive drugs, such as mycophenolate mofetil or calcineurin inhibitors. These drugs can damage the intestinal mucosa, leading to impaired absorption of vitamins [17,61]. Additionally, glucocorticoids, often used in the first months after transplantation, can affect protein and vitamin metabolism, increasing the risk of deficiencies. Another factor is kidney dysfunction, which is a common complication of immunosuppressive therapy and can disrupt the metabolism of B vitamins, especially B6 and B12. Many studies also highlight the role of protein-caloric malnutrition, which often occurs in patients in the perioperative period and in the first months after transplantation, when energy requirements are increased and appetite and food tolerance may be reduced [62].

Deficiency of B vitamins can lead to numerous complications, which are particularly important in heart transplant recipients. One of the most important is hyperhomocysteinemia, which is closely related to a deficiency of folic acid, vitamin B6, and vitamin B12. Homocysteine is an amino acid formed as a result of methionine metabolism, and its proper metabolism requires the presence of B vitamins as enzymatic cofactors. International studies have shown that heart transplant patients with vitamin B6, B9, and B12 deficiency have significantly higher homocysteine concentrations, which may increase the risk of cardiovascular complications such as TVD, thrombosis, or endothelial dysfunction and increases the risk of thrombotic complications. This mechanism results from increased oxidative stress, activation of pro-inflammatory processes and disorders in the coagulation system, which makes B vitamin deficiencies an important, modifiable risk factor in heart recipients [63]. TVD is one of the most serious late complications after heart transplantation and is characterized by diffuse thickening of the inner membrane of the coronary vessels of the transplanted heart [64]. A number of studies have shown that B vitamin deficiency can contribute to the progression of TVD by increasing oxidative stress, increasing inflammation and impaired endothelial function. Although these data are not conclusive, and some studies do not confirm a direct link between B vitamin deficiency and the progression of TVD, the role of these vitamins as potential modulators of inflammatory and metabolic processes remains the subject of intensive research [17,61,62].

Deficiency of B vitamins can also affect the functioning of the nervous system, which is especially important in heart transplant recipients, who often struggle with neuropathies, cognitive impairment, and depressive symptoms [60]. Studies have shown that vitamin B12 deficiency can lead to peripheral neuropathy, balance disorders, muscle weakness, and cognitive decline. In heart transplant patients, these symptoms may be difficult to distinguish from the side effects of immunosuppressive drugs such as tacrolimus, which can cause tremors, neuropathies, and neurological disorders. Vitamin B1 (thiamine) deficiency can lead to Wernicke’s encephalopathy, although this is a rare complication [17,61,62,63]. However, in patients with severe malnutrition or malabsorption this risk may be increased. Vitamin B6 deficiency can lead to seizures, neuropathy, and mood disorders, which can also affect the quality of life of transplant recipients. B vitamins also play an important role in the functioning of the immune system. In vitro studies have shown that vitamin B6 affects lymphocyte proliferation, cytokine production, and NK cell function [60].

Vitamin B6 deficiency can lead to a weakened immune response, which is especially important in heart transplant recipients who are at risk of infections due to the use of immunosuppression. Several clinical trials have shown that patients with B vitamin deficiency have a higher incidence of infections, especially respiratory tract infections, and opportunistic infections, although these data are limited and require further analysis [64]. This association is biologically plausible, as vitamins B6, B9, and B12 are essential cofactors in nucleotide synthesis, lymphocyte proliferation, and the maintenance of normal cellular immunity. Deficiencies in these vitamins can impair both humoral and cell-mediated immune responses, leading to reduced activity of NK cells, impaired maturation of T lymphocytes, and diminished synthesis of immunoglobulins [63].

In heart transplant recipients, who are already exposed to chronic immunosuppression, even mild disturbances in these pathways may further compromise host defense mechanisms. Moreover, hyperhomocysteinemia—a hallmark of B vitamin deficiency—has been shown to promote endothelial dysfunction and low-grade inflammation, which may additionally weaken mucosal barriers and facilitate pathogen invasion. Although current evidence is not yet sufficient to establish a causal relationship, preliminary findings suggest that monitoring and correcting B vitamin deficiencies may have broader clinical implications beyond cardiovascular protection, potentially contributing to improved immune resilience in this vulnerable population [64]. It is possible that a deficiency of B vitamins acts as a marker of general malnutrition and weakness of the body, rather than as a direct factor that increases the risk of infection. An excess of B vitamins, although much rarer than their deficiency, can also be a clinical problem. This is most often the case with vitamin B6, the excessive supplementation of which can lead to sensory neuropathy. Studies have shown that patients taking high doses of vitamin B6 for a long time may experience numbness, tingling, imbalance, and muscle weakness [62,64]. In heart transplant recipients, these symptoms may be difficult to distinguish from the side effects of immunosuppressive drugs, which can lead to a delay in diagnosis.

Excess vitamin B3 (niacin) can lead to liver damage, redness of the skin, and metabolic disorders, which is especially important in transplant patients who often have liver dysfunction associated with immunosuppressive therapy [61]. Excess vitamin B9 (folic acid) can mask vitamin B12 deficiency, which can lead to progression of neuropathy and neurological disorders. For this reason, supplementation of B vitamins in heart transplant recipients should be carried out carefully and under control of the concentration of individual vitamins. Interactions of B vitamins with immunosuppressive drugs are another important area of research. It has been shown that calcineurin inhibitors can affect the metabolism of B vitamins by affecting kidney function and enzymes involved in their metabolism. Mycophenolate mofetil may interfere with intestinal absorption, which may lead to vitamin B9 and B12 deficiency [60,62,63,64].

Glucocorticoids can affect the metabolism of proteins and vitamins, increasing the risk of deficiencies. Several studies have shown that patients treated with tacrolimus have lower levels of vitamin B6 and B12 than patients treated with ciclosporin, although these data are inconclusive and may depend on kidney function and the dose of medication. In the context of therapeutic options, supplementation of B vitamins is often used in heart transplant recipients, especially in patients with hyperhomocysteinemia or laboratory-confirmed deficiencies [65]. Many centers routinely supplement folic acid and vitamin B12 are used in patients with renal dysfunction, as these deficiencies are common and can lead to metabolic complications [61,64]. However, there are no large randomized trials that would unequivocally confirm the effect of B vitamin supplementation on the clinical prognosis in heart transplant recipients. Several smaller studies have shown that B vitamin supplementation can improve endothelial function, reduce homocysteine levels, and improve metabolic parameters, but these data are limited and require further analysis [66].

The international literature emphasizes the need for further research on the role of B vitamins in heart transplant recipients. There is a lack of large randomized trials assessing the effect of supplementation on the risk of TVD, transplant rejection, or metabolic complications. Research is also needed on the interactions of B vitamins with immunosuppressive drugs and on the role of these vitamins as biomarkers of nutritional status and metabolic function. It is still not known whether B vitamins are causal factors or only risk markers, but their importance in transplantology remains indisputable [62,67].

## 4. Other Prevalent Macro- and Micronutrient Deficiencies in Heart Transplant Recipients

Table 3 presents key information on the most common macro- and micronutrient deficiencies in heart transplant recipients, highlighting their complex etiology and clinical significance. These data reflect the multifactorial nature of eating disorders in this population, resulting both from the physiological consequences of severe heart failure and transplantation. They also arise from the long-term use of immunosuppressive drugs, which affect the metabolism, absorption and excretion of many nutrients. Deficiencies of protein, vitamin D, B vitamins, and electrolytes such as magnesium or potassium are particularly important, as they can directly affect the functioning of the immune system, heart rhythm stability, and bone health, as well as the patient’s overall physical performance. It is worth noting that some deficiencies—especially magnesium, potassium or vitamin D—are closely related to the side effects of immunosuppressive drugs, such as tacrolimus, cyclosporine, or glucocorticoids. Others, such as iron, B vitamins, and zinc deficiencies, may result from chronic inflammation, intestinal absorption disorders, or increased metabolic demand [60]. The clinical consequences of these disorders are multidimensional and include both metabolic and immune complications, which highlights the need for regular nutritional assessment and monitoring of biochemical parameters. The table also indicates the importance of individualization of nutritional behavior. Depending on the type of deficiency, it may be necessary to increase dietary supply, oral or intravenous supplementation, and in some cases, modify immunosuppressive therapy. A comprehensive approach to the assessment and correction of nutritional deficiencies is therefore a key component of the care of heart transplant recipients, affecting not only their general condition but also the long-term survival and function of the transplanted organ [17,61].

Another important aspect that should be emphasized in the context of nutritional deficiencies in heart transplant recipients is the dynamic nature of metabolic changes occurring both in the perioperative period and in long-term follow-up [62]. The early period after transplantation is characterized by increased catabolism, increased energy demand and significant oxidative stress, which promotes the rapid depletion of macro- and micronutrient reserves [63]. At this time, deficiencies of protein, electrolytes, and fat-soluble vitamins are particularly common, which can exacerbate wound healing disorders, weaken muscle function, and increase susceptibility to infections. On the other hand, in the later period, deficiencies resulting from chronic use of immunosuppressive drugs, intestinal absorption disorders, and changes in body composition, such as sarcopenia or celiac obesity, dominate. Later in the transplant period, deficiencies resulting from chronic use of immunosuppressive drugs, intestinal absorption disorders, and changes in body composition, such as sarcopenia or visceral obesity, become increasingly important. Long-term immunosuppression affects not only the metabolism of many micronutrients, but also the functioning of the digestive tract, leading to impaired absorption of vitamins and trace elements. At the same time, progressive sarcopenia limits the body’s metabolic reserves, and visceral obesity promotes chronic inflammation and hormonal disorders, which further exacerbates the risk of deficiencies. As a result, patients in the distant post-transplant period are dominated by complex, overlapping eating disorders that may affect immunity, treatment tolerance, and overall clinical status [62,64].

Among macronutrients, protein is of particular importance, the deficiency of which is one of the most frequently observed disorders in this population. In heart transplant recipients, insufficient protein supply may result from both dietary restrictions and increased glucocorticoid-induced catabolism and chronic inflammation. Clinical consequences include loss of muscle mass, weakening of muscle strength, deterioration of exercise tolerance and an increased risk of infection. In clinical practice, it is particularly important to monitor parameters such as albumin, prealbumin, or CRP, which, although limited, can help assess nutritional status and responses to nutritional interventions [65].

Among micronutrients, one of the most frequently deficient is magnesium. Hypomagnesemia in heart transplant recipients is highly common, mainly due to tacrolimus and ciclosporin, which increase the excretion of magnesium by the kidneys. This deficiency can lead to cardiac arrhythmias and neuromuscular hyperactivity, as well as increase the risk of nephrotoxicity of calcineurin inhibitors [66]. Importantly, hypomagnesemia can also exacerbate hypokalemia, which further increases the risk of ventricular arrhythmias. For this reason, routine monitoring of magnesium levels and magnesium supplementation are a key part of post-transplant care [67].

A similarly important problem is potassium deficiency, which can result from both the effects of immunosuppressive drugs and diuretics used to treat concomitant heart failure or hypertension. Hypokalemia can lead to arrhythmias, muscle weakness, and increased sensitivity to the toxic effects of digitalis glycosides [68]. In clinical practice, it is necessary not only to monitor potassium levels, but also to assess acid–base balance and kidney function, which may affect its economy. Vitamin D is another key micronutrient that is common in heart transplant recipients. This is due to both limited exposure to sunlight and intestinal absorption disorders, as well as the effect of glucocorticoids on calcium and bone metabolism [69]. Vitamin D deficiency is associated with the risk of osteopenia, osteoporosis, increased fracture susceptibility, as well as immune disorders. In the heart transplant population, early supplementation and monitoring of 25(OH)D levels are particularly important, especially in patients taking high doses of steroids [70]. B vitamins, especially B6, B12, and folic acid, are also often deficient. Their reduced concentrations may result from malabsorption disorders, increased metabolic demand, and chronic inflammation. These deficiencies can lead to hyperhomocysteinemia, which is a risk factor for cardiovascular complications, as well as hematological and neurological disorders [67,69]. In clinical practice, it is important to regularly monitor homocysteine and B vitamin levels, especially in patients with symptoms of neuropathy or anemia. Iron is another micronutrient of great clinical importance. Iron deficiency in heart transplant recipients can result from chronic inflammation, blood loss, malabsorption, or increased demand [71]. This deficiency leads to anemia, weakness, impaired exercise tolerance, and an increased risk of hospitalization. In clinical practice, it is necessary to differentiate iron deficiency from anemia of chronic diseases and to select the appropriate form of supplementation—oral or intravenous. Zinc and selenium are other elements whose deficiencies can have significant clinical consequences [72,73,74]. Zinc plays a key role in wound healing, immune system function and protein metabolism. Its deficiency can lead to taste disorders, loss of appetite, delayed wound healing, and increased susceptibility to infections. Selenium, on the other hand, is essential for the functioning of antioxidant enzymes, such as glutathione peroxidase. Selenium deficiency can increase oxidative stress, affect heart muscle function, and increase the risk of infectious complications [69,71].

To sum up, macro- and micronutrient deficiencies in heart transplant recipients represent a complex clinical problem, requiring a multidimensional diagnostic and therapeutic approach [75]. Regular assessment of nutritional status, monitoring of biochemical parameters, and individualization of nutritional interventions are crucial for improving the prognosis and quality of life of transplant patients [76]. The inclusion of a clinical dietitian in the transplant team, as well as close cooperation between specialists, allow for effective prevention and treatment of deficiencies, which translates into better functioning of the transplanted organ and a reduced risk of metabolic and immune complications [77,78,79].

## 5. Risk Factors for Micronutrient Deficiency in Heart Transplant Patients

Heart transplant patients are a population particularly vulnerable to developing micronutrient deficiencies, which results from overlapping factors related to the preoperative, perioperative and chronic immunosuppression periods. Even before the procedure, many patients experience advanced heart failure leading to cachexia, sarcopenia, decreased appetite, and impaired intestinal absorption secondary to visceral hypoperfusion [61,70]. This condition promotes deficiencies in B vitamins, vitamin D, iron, zinc, and selenium, which can persist or worsen after transplantation. In addition, chronic use of drugs used in heart failure, especially loop diuretics, leads to the loss of electrolytes and micronutrients, such as magnesium, potassium, or zinc, and therapy with aldosterone antagonists can disrupt sodium potassium metabolism and affect magnesium metabolism [80]. The perioperative period is associated with an intense inflammatory response and metabolic stress, which increase the consumption of antioxidants such as vitamin C, vitamin E, or selenium, and elements involved in repair processes, including zinc and copper. In the first few days after transplantation, patients often require enteral or parenteral nutrition, and limited oral intake resulting from intubation, impaired consciousness, or nausea can lead to an insufficient supply of vitamins and trace elements, especially if the mixtures used are not properly balanced [56].

A key risk factor for micronutrient deficiencies in the postoperative period is chronic immunosuppression. Glucocorticoids increase calcium excretion, disrupt vitamin D metabolism, impair the absorption of calcium and magnesium, and promote the development of osteopenia and osteoporosis. Calcineurin inhibitors such as ciclosporin and tacrolimus may lead to nephrotoxicity, calcium-phosphate disorders, and hypomagnesemia due to impaired renal reabsorption. Antimetabolites, including mycophenolate mofetil and azathioprine, often induce diarrhea and malabsorption, resulting in the loss of zinc, selenium, and water-soluble vitamins [81]. Gastrointestinal disorders are another important element that increases the risk of deficiencies. Diarrhea, intestinal dysbiosis, and opportunistic infections such as CMV infection or *Clostridioides difficile* lead to loss of electrolytes, B vitamins, zinc, and selenium, and impaired absorption of fats, and thus vitamins A, D, E, and K. Gastrointestinal motility disorders, including gastroparesis, nausea, and vomiting, further limit food intake and can lead to energy and micronutrient deficiencies. After transplantation, obesity and metabolic syndrome often develop, partially induced by chronic steroid therapy. They increase oxidative stress and the need for antioxidants, can disrupt iron metabolism and increase the need for B vitamins. Nephrotoxicity of calcineurin inhibitors can lead to vitamin D metabolism disorders, hyperphosphatemia or hypophosphatemia, and calcium-magnesium metabolism disorders, which further increases the risk of deficiencies [17,73,77].

Dietary and behavioral factors also play an important role. Patients often follow elimination or restrictive diets, fearing drug interactions or the risk of infections associated with eating fresh products [82]. This can lead to deficiencies in vitamin C, folate, iron, selenium, and other micronutrients. Insufficient nutritional education after transplantation further increases the risk of insufficient supply of micronutrients, especially in the context of increased metabolic demand. Drug–nutrient interactions are also important. Proton pump inhibitors can reduce the absorption of magnesium, iron, and vitamin B12, while bile acid binders limit the absorption of fat-soluble vitamins. Steroids and calcineurin inhibitors affect the metabolism of vitamin D and calcium, and some immunosuppressive drugs increase the need for antioxidants [64,67,73].

All of these factors put heart transplant patients at multifactorial risk of micronutrient deficiencies. Early identification of these risks, regular assessment of nutritional status, and targeted supplementation are key elements of comprehensive care for transplant recipients and can significantly improve their prognosis and quality of life [70,73,83]. Table 4 summarizes the risk factors for micronutrient deficiencies in heart transplant patients.

## 6. Limitations and Future Research

This review has several important limitations that need to be taken into account when interpreting the results. First, the available literature on micronutrient deficiencies in heart transplant recipients is limited and has considerable heterogeneity. Differences in study design, sample size, post-transplant follow-up time, and diagnostic criteria used make it difficult to compare results directly and may lead to an underestimation or overestimation of the actual incidence of deficiencies. Many studies have involved small groups of patients, often from individual centers, which limits the possibility of generalizing the results to a wider population of transplant recipients.

Second, most of the available work focused on selected micronutrients—mainly iron, vitamin D, and B vitamins—while data on other relevant elements, such as selenium, zinc, copper, iodine, and fat-soluble vitamins, remain fragmentary or even absent. The lack of comprehensive analyses including a full micronutrient profile makes it difficult to assess the scale of the problem and identify potential interactions between deficiencies.

Third, studies have used different definitions of micronutrient deficiencies, often based on criteria developed for the general population or patients with heart failure, which may not reflect specific metabolic changes in transplant recipients. For example, iron deficiency criteria differed between studies, and reference values for vitamin D were not uniformly applied. The lack of diagnostic standardization is a significant barrier to the interpretation of results and the development of consistent clinical recommendations.

Fourth, according to the scoping review methodology, no formal assessment of the methodological quality of the included studies was conducted. While this approach allows for a broad mapping of available data, it limits the ability to assess the strength of evidence and the risk of bias. In addition, the influence of publication bias cannot be ruled out—studies showing significant deficiencies or clinical associations may be published more frequently than those with neutral results.

Finally, few studies have evaluated the clinical effects of micronutrient supplementation in heart transplant recipients. Data on the effectiveness of interventions, optimal doses, duration of therapy, and safety are limited, making it impossible to develop unambiguous therapeutic recommendations. There is also a lack of studies analyzing the long-term impact of deficiency correction on survival, transplant function, risk of infection, or the development of metabolic complications. In conclusion, while the available data clearly indicate that micronutrient deficiencies are common and clinically relevant in heart transplant recipients, further well-designed studies are needed to develop consistent, evidence-based guidelines for the diagnosis, monitoring, and treatment of these disorders.

The review was conducted using the most frequently used databases and did not include the gray literature.

## 7. Conclusions

Micronutrient deficiencies in heart transplant recipients are a significant and still under-studied clinical problem that may affect immune function, immunosuppressive drug metabolism, infection risk, and long-term prognosis. The results of this review indicate that the most commonly reported disorders are deficiencies of iron, vitamin D, and B vitamins, but the available data suggest that other micronutrients—such as zinc, selenium, and iodine—may also play an important role in the health of this population. The heterogeneity of methodological research, the lack of uniform diagnostic criteria, and the limited number of prospective analyses make it difficult to accurately assess the scale of the problem. The evidence gathered highlights the need for routine monitoring of selected micronutrients in heart transplant care, especially in the first months after surgery and in patients with chronic immunosuppression, inflammation, or concomitant renal dysfunction. Early identification and correction of deficiencies can contribute to improving treatment tolerance, reducing the risk of complications and improving the quality of life of patients. At the same time, the results of the review indicate an urgent need for further research—particularly prospective, multicenter analyses covering a wide panel of micronutrients and interventional studies to assess the effectiveness of supplementation. The development of standardized diagnostic criteria and management algorithms could significantly improve the care of heart transplant recipients and contribute to improving their long-term prognosis.

## Figures and Tables

**Table 1:** Summary of search strategy.

Database	Date of Search	Search String/Controlled Vocabulary	Filters Applied
PubMed	10 January 2026	(“Micronutrients”[MeSH] OR micronutrient* OR vitamin* OR “trace element” OR “mineral deficiency”) AND (“Heart Transplantation”[MeSH] OR “heart transplant” OR “cardiac transplant” OR “orthotopic heart transplant” OR HTx) AND (“Deficiency Diseases”[MeSH] OR deficiency OR deficiencies)	English; Humans; Adults (≥18 years); Full text; Article types: Clinical Study, Observational Study, RCT, Review, Systematic Review, Meta-analysis
Scopus	12 January 2026	TITLE-ABS-KEY (micronutrient* OR vitamin* OR “trace element” OR “mineral deficiency”) AND TITLE-ABS-KEY (“heart transplant” OR “cardiac transplant*” OR HTx)	English; Article; Review; Adult
Web of Science	14 January 2026	TS = (micronutrient* OR vitamin* OR “trace element” OR deficiency) AND TS = (“heart transplant” OR “cardiac transplant*”)	English; Document types: Article, Review; Adult
EBSCO (Medline Complete)	15 January 2026	(MH “Micronutrients” OR micronutrient* OR vitamin* OR “trace element”) AND (MH “Heart Transplantation” OR “heart transplant”) AND (deficiency OR deficiencies)	English; Peer-reviewed; Full text; Adults
Cochrane Library	17 January 2026	micronutrient* OR vitamin* OR “trace element” AND “heart transplant”	English; Trials; Reviews
Google Scholar	20 January 2026	“micronutrient deficiency” AND “heart transplant”	English; First 200 results screened

**Table 2:** Characteristics and findings of studies included in this review.

Author, Year	Country	Aim of the Study	Participants	Age and Gender	Results and Findings
Brautaset Englund K. et al., 2022 [12]	Norway	Explore determinants of iron status in the 102 IronIC participants to better define iron deficiency in the HTx population	102 HTx recipients	- between 18 and 80 years old (average age: 55 years) - men: 64 (66%), women: 33 (34%)	✔ HTx recipients with iron deficiency as defined in heart failure do not have elevated hepcidin levels, although inflammatory markers are modestly increased ✔ The high ferritin cut-offs used in heart failure may not be suitable to define iron deficiency in the HTx population ✔ Hepcidin and sTfR should be measured to identify patients with true iron deficiency, who might benefit from treatment with intravenous iron
V Brautaset Englund K. et al., 2021 [13]	Norway	To evaluate the prevalence and determinants of iron deficiency in Norwegian heart transplant recipients	378 heart transplant recipients	- Age: 56 ± 15 - 274 men (72%), 104 women (28%)	✔ Iron deficiency defined as ID_HF_, ID_F100_, or ID_Tsat_ is prevalent in the heart transplant population, while ID_F30_ is not ✔ Further research is required to identify the mechanisms of iron homeostasis in heart transplant recipients and to establish a definition of iron deficiency suitable for this population ✔ C-reactive protein was independently associated with ID_HF_ and ID_Tsat_
Przybyłowski P. et al., 2011 [14]	Poland	Assess the prevalence of functional iron deficiency in heart and kidney transplant recipients based on data from recent medical records	169 prevalent heart allograft recipients (35 females)	- 169 prevalent heart allograft recipients (35 females) - Age: 52.92 ± 14.69	✔ Absolute iron deficiency was observed in 35% of the heart ✔ Functional iron deficiency occurred in 4% of the heart ✔ Functional iron deficiency was associated with significantly higher serum ferritin and lower TSAT ✔ Heart transplant recipients with absolute iron deficiency showed lower erythrocyte counts
Stein E.M. et al., 2011 [15]	USA	Review the prevalence of vitamin D deficiency in organ transplant candidates and in long-term transplant recipients Summarize interventional trials evaluating vitamin D, 1,25(OH)_2_D, and its analogues for the prevention and treatment of bone loss following solid organ transplantation	candidates for transplantation of various organs (heart, lungs, liver, kidneys); transplant recipients—both in the early period and in long-term follow-up; patients with diseases leading to organ failure	N/A (not applicable)	✔ Although several studies have demonstrated that active forms of vitamin D and its analogues prevent bone loss following transplantation, the data do not show consistent benefit ✔ Demographic and lifestyle factors are important in determining D status in transplant recipients ✔ Worse vitamin D status is associated with poorer general health, lower albumin, and even decreased survival among these patients ✔ Vitamin D deficiency is prevalent among patients with end-stage organ failure awaiting transplant ✔ Low serum 25-hydroxyvitamin D (25-OHD) levels in these patients may be related to many disease-specific factors, as well as decreased sunlight exposure and limited intake of foods containing vitamin D
Stein E.M. et al., 2009 [16]	USA	Evaluate and directly compare the prevalence of vitamin D insufficiency in cardiac or liver transplant recipients at the time of organ transplantation	46 heart transplant recipients	- mean age 53 (range 22–72) - 56 men (81%) and 13 women (19%)	✔ Vitamin D deficiency is highly prevalent among heart and liver transplant recipients ✔ As vitamin D deficiency has many serious skeletal and extra-skeletal sequelae, physicians who treat transplant patients should maintain a high degree of vigilance for this problem
Nahlawi M. et al., 2002 [17]	USA	The role of a high plasma total homocysteine and low levels of B vitamins as risk factors for atherothrombotic outcomes in heart transplant patients	160 patients who underwent orthotopic cardiac transplantation	- 127 men and 33 women - mean age 49 ± 11 years	✔ An elevated plasma total homocysteine level and low folate and vitamin B6 concentrations have been identified as risk factors for atherosclerosis and thrombosis ✔ Cardiovascular complications are major causes of death and morbidity in heart transplant recipients ✔ Authors prospectively evaluated the role of a high plasma total homocysteine and low levels of B vitamins as risk factors for atherothrombotic outcomes in heart transplant patients
Wozniak-Grygiel E. et al., 2009 [18]	Poland	Assess urinary iodine and thyroid gland hormone management among a Polish population of heart transplant recipients	32 heart transplant recipients	- 26 men and 6 women - average age: 50.4 ± 12.6	✔ There exists significant iodine deficiency among heart transplant recipients ✔ Measurements of urinary iodine together with thyroid gland hormones may be essential to prevent thyroid gland disturbances in these patients
Małyszko J. et al., 2012 [19]	Poland	Summarize the current knowledge on iron metabolism in kidney, heart, and liver transplant recipients	-	N/A	✔ Iron deficiency and/or anemia, as well as iron overload, are frequently observed but the precise mechanism has not been fully elucidated ✔ Iron status check-up should be a part of long-term follow-up because disturbances in iron metabolism are a possible risk factor of infections and mortality in solid transplant recipients ✔ Regular evaluation of iron metabolism should be a mandatory element of transplant care
Przybyłowski P. et al., 2018 [20]	Poland	Assess vitamin D concentration in patients after heart and kidney transplantation	98 stable heart transplant recipients were enrolled in the study	- 74 men, 24 women - Age: 54.3 ± 13.2 years	✔ Vitamin D deficiency is more common in patients after heart transplantation than in kidney allograft recipients despite similar kidney function ✔ The possible associations between the cardiovascular system and vitamin D merit further studies
Przybyłowski P. et al., 2013 [21]	Poland	Assess whether hepcidin was related to functional iron deficiency among orthotopic heart transplant (OHT) recipients treated with mammalian target of rapamycin (mTOR) antagonist (n = 35)	The study included 169 patients including 35 females who underwent their first OHT	- 127 men and 33 women, - mean age 49 ± 11 years	✔ Functional iron deficiency which is common among OHT patients treated with mTOR, was associated with high hepcidin levels and inflammatory markers ✔ This form of anemia in mTOR-treated OHT resembles the disorder of chronic disease, suggesting that OHT patients show low-grade inflammation, which should be investigated for underlying, potentially reversible causes. ✔ Iron treatment should also be considered
Thiem U. et al., 2013 [22]	Austria	Crystallizes and summarizes existing data on the status quo of vitamin D deficiency in patients with organ failure and in solid organ transplant recipients	-	N/A	✔ Vitamin D deficiency is extremely common in patients with organ failure ✔ Vitamin D deficiency very often persists even after transplantation ✔ The causes of this deficiency are multifactorial ✔ There are no clear recommendations for the treatment of vitamin D deficiency in patients with organ failure
Przybylowski P. et al., 2016 [23]	Poland	Determine prevalence of absolute and functional iron deficiency in patients with heart failure (n = 269) and after heart transplantation (n = 130) and their relation to parameters of iron status and inflammation	130 patients who underwent their first orthotopic heart transplantation (OHT)	- 32 women, 98 men - age: 54.54 ± 13.98	✔ Both absolute and functional iron deficiency were present in a considerable group of patients ✔ This population should be carefully screened for possible reversible causes of inflammation

**Table 3:** Key information on the most common macro- and micronutrient deficiencies in heart transplant recipients [17,60,61,62,63,64,65,66,67,68,69,70,71,72,73,74,75,76,77,78,79].

Component	Incidence of Deficiency in Transplant Recipients	Main Causes	Clinical Consequences	Management/Prevention
Protein	High (20–40%)	Perioperative malnutrition, postoperative catabolism, infections, steroid therapy	Muscle mass loss, weakened immunity, delayed wound healing	Nutritional status assessment, increased protein intake, dietary support
Energy (calories)	High	Reduced appetite, nausea, malabsorption, increased metabolic demand	Weight loss, weakness, poorer rehabilitation tolerance	Individualized diet, oral or enteral nutrition
Vitamin D	Very high (70–90%)	Limited sun exposure, renal failure, steroid therapy	Osteopenia, osteoporosis, infections, immune disorders	Supplementation, 25(OH)D monitoring
B vitamins (B6, B9, B12)	High (30–60%)	Malabsorption, immunosuppressants, renal failure	Hyperhomocysteinemia, neuropathies, anemia, cognitive impairment	Targeted supplementation, homocysteine level assessment
Vitamin C	Moderate	Oxidative stress, increased demand, insufficient supply	Weakened immunity, poorer wound healing	Diet rich in vegetables and fruits, supplementation
Vitamin A	Rare but possible	Fat malabsorption, liver failure	Visual disturbances, weakened immunity	Monitoring, supplementation as needed
Iron	Moderate to high	Perioperative bleeding, chronic inflammation, renal failure	Anemia, weakness, poorer exercise tolerance	Oral or intravenous supplementation, anemia diagnosis
Magnesium	High (especially with tacrolimus)	Immunosuppressants (tacrolimus, cyclosporine), diuretics	Heart rhythm disturbances, muscle spasms, weakness	Supplementation, electrolyte monitoring
Potassium	Variable (both deficiency and excess)	Diuretics, calcineurin inhibitors, renal failure	Heart rhythm disturbances, muscle weakness	Regular monitoring, dietary adjustments
Phosphorus	Moderate	Renal failure, malabsorption	Muscle weakness, impaired bone mineralization	Phosphate control, supplementation or restriction
Zinc	Moderate	Malabsorption, increased demand, immunosuppression	Taste disturbances, poorer wound healing, immune impairment	Supplementation, zinc-rich diet
Selenium	Moderate	Oxidative stress, renal failure, malnutrition	Immune weakness, heart disorders	Supplementation if deficiency occurs
Omega-3 fatty acids	Moderate	Insufficient supply, malabsorption	Lipid disorders, inflammation, risk of CAV	Diet rich in fish, EPA/DHA supplementation

**Table 4:** Risk factors for micronutrient deficiencies in heart transplant patients [61,70,73,77,83].

Risk Factor Category	Mechanism/Description	Most Commonly Affected Micronutrients
Pre-transplant status: cachexia, sarcopenia, malnutrition	Reduced intake, impaired absorption, increased metabolic demand	Vitamins B1, B6, B12, D; iron; zinc; selenium
Chronic pre-transplant medication use (diuretics, aldosterone antagonists)	Loss of electrolytes and trace elements, mineral metabolism disorders	Magnesium, potassium, zinc
Perioperative metabolic and inflammatory stress	Increased consumption of antioxidants and repair elements	Vitamins C, E; selenium; zinc; copper
Limited oral intake after surgery	Intubation, nausea, incomplete enteral/parenteral nutrition	All vitamins and trace elements, especially vitamin D, folate, iron
Glucocorticosteroids	Increased calcium excretion, impaired vitamin D metabolism, insulin resistance	Calcium, magnesium, vitamin D
Calcineurin inhibitors (tacrolimus, cyclosporine)	Nephrotoxicity, magnesium loss, impaired calcium-phosphate metabolism	Magnesium, calcium, phosphorus, vitamin D
Antimetabolites (MMF, azathioprine)	Diarrhea, malabsorption	Zinc, selenium, vitamin B-complex, fat-soluble vitamins
Diarrhea, dysbiosis, intestinal infections (CMV, *C. difficile*)	Loss of electrolytes and vitamins, impaired fat absorption	Vitamins A, D, E, K; vitamin B; zinc; selenium
Gastrointestinal motility disorders (gastroparesis, nausea)	Restricted food intake	All micronutrients, especially vitamin B and iron
Obesity and metabolic syndrome after transplantation	Increased oxidative stress, impaired iron metabolism	Antioxidants (vitamins C, E, selenium), vitamin B-complex, iron
Kidney failure after transplantation	Impaired vitamin D, phosphate, and magnesium metabolism	Vitamin D, calcium, phosphorus, magnesium
Dietary restrictions and low nutritional awareness	Elimination of fresh produce, monotonous diet	Vitamin C, folate, iron, selenium
Drug–nutrient interactions (PPIs, bile acid-binding resins)	Reduced vitamin and mineral absorption	Magnesium, iron, vitamin B12, vitamin A/D/E/K

## Data Availability

No new data were created or analyzed in this study. Data sharing is not applicable to this article.

## Abbreviations

The following abbreviations are used in this manuscript:

PCC	Population–Concept–Context
DNA	deoxyribonucleic acid
PRISMA-ScR	preferred reporting items for systematic reviews and meta-analyses for scope reviews
JBI	Joanna Briggs Institute
n	number
HTx	heart transplantation
ID	iron deficiency
TSAT	transferrin saturation
25-OHD	25-hydroxyvitamin D
OHT	orthotopic heart transplant
VDRs	vitamin D receptors
CMV	Cytomegalovirus
EBV	Epstein–Barr Virus
TVD	transplant vascular disease
CAV	cardiac allograft vasculopathy
IL-6	Interleukin-6
TNF	Tumor Necrosis Factor-alpha
B9	folic acid
Thiamine	B1
Riboflavin	B2
Niacin	B3
Biotin	B7
NK	natural killer cells
CRP	C-reactive protein
EPA	eicosapentaenoic acid
DHA	docosahexaenoic acid
PPIs	proton pump inhibitors
mTOR	mammalian target of rapamycin
